# 

**Published:** 1807-10

**Authors:** 


NEW MEDICAL PUBLICATIONS.

A Treatise on the Diseases of the Joints; being the Observations for which the Prize for 1806 was adjudged by the Royal College of Snrgeons in London; by Samuel Cooper. 5s. 8vo. bound.

A Popular View of Vaccine Inoculation, with the Practical Mode of Conducting it, shewing the Analogy between the Small-Pox, and the Cow-Pox, and the Advantages of the latter; by Joseph Adams. 4s. 6d. boards.

